# NeuralLasso: Neural Networks Meet Lasso in Genomic Prediction

**DOI:** 10.3389/fpls.2022.800161

**Published:** 2022-04-29

**Authors:** Boby Mathew, Andreas Hauptmann, Jens Léon, Mikko J. Sillanpää

**Affiliations:** ^1^Bayer CropScience, Monheim am Rhein, Germany; ^2^Institute of Crop Science and Resource Conservation, University of Bonn, Bonn, Germany; ^3^Research Unit of Mathematical Sciences, University of Oulu, Oulu, Finland; ^4^Department of Computer Science, University College London, London, United Kingdom

**Keywords:** neural networks, LASSO, local epistasis, genomic selection, whole genome prediction

## Abstract

Prediction of complex traits based on genome-wide marker information is of central importance for both animal and plant breeding. Numerous models have been proposed for the prediction of complex traits and still considerable effort has been given to improve the prediction accuracy of these models, because various genetics factors like additive, dominance and epistasis effects can influence of the prediction accuracy of such models. Recently machine learning (ML) methods have been widely applied for prediction in both animal and plant breeding programs. In this study, we propose a new algorithm for genomic prediction which is based on neural networks, but incorporates classical elements of LASSO. Our new method is able to account for the local epistasis (higher order interaction between the neighboring markers) in the prediction. We compare the prediction accuracy of our new method with the most commonly used prediction methods, such as BayesA, BayesB, Bayesian Lasso (BL), genomic BLUP and Elastic Net (EN) using the heterogenous stock mouse and rice field data sets.

## Introduction

The introduction of Genomic Selection (GS) (Meuwissen et al., 2001) along with the availability of low cost genotyping platforms has resulted in a major paradigm shift in both animal and plant breeding. Since then, GS has been successfully applied for efficient selection and accelerating the breeding process in various breeding programs (Spindel et al., 2015; Garner et al., 2016; Hickey et al., 2017; Voss-Fels et al., 2019). Even though GS has now been widely implemented in practice, still considerable effort has been given to improve the prediction accuracy in GS beyond the current limits. Various factors can affect the prediction accuracy in GS including marker density, heritability of the trait, population size, constitution of the learning population and the statistical model used to predict the genomic breeding values (Meuwissen, 2009; Liu et al., 2018; Norman et al., 2018). Recently many studies tried to incorporated the transcriptome data (Li et al., 2019; Azodi et al., 2020) into genomic prediction models, in order to improve the prediction accuracy in GS.

The genomic prediction models can be divided roughly into two classes: (1) genomic best linear unbiased prediction (GBLUP) based on linear mixed models and (2) the whole-genome regression (WGR) based on multilocus regression models. In the first approach, the genetic background of the trait is assumed to be polygenic while in the latter, more oligogenic genetic background is assumed. Again in the first, molecular markers are used to construct the genomic relationship matrix while in the latter, molecular markers represent considered set of regression variables in the model. However, note that WGR model can be written also as the GBLUP model with a trait-specific relationship matrix having own variance component for each SNP in the diagonal (Zhang et al., 2010; Piepho et al., 2012; Resende et al., 2012; Shen et al., 2013).

Epistasis (genetic interaction) is one of the major reason for the non-linearity in the genotype-phenotype relationship and considerable efforts have been given to model epistasis in genomic prediction models (Hu et al., 2011; Wittenburg et al., 2011; Wang et al., 2012; Jiang and Reif, 2015). Recently, many studies even pointed out the importance of local epistasis (interactions that span short segments of the genome) (Wei et al., 2014; Akdemir and Jannink, 2015; Akdemir et al., 2017; He et al., 2017; Liang et al., 2020). Although it is well known that epistasis (both local and global) interactions contribute to many complex traits (Taylor and Ehrenreich, 2014, 2015; Albert and Kruglyak, 2015), most of the genomic prediction models account for the pair-wise interactions due to the computational complexity of screening through all possible combinations.

Most of the WGR models used in GS are based on linear regression procedure and have been successfully adopted to predict complex phenotype in plant and animal breeding programs (Meuwissen et al., 2001; Park and Casella, 2008; Mathew et al., 2019). Nonlinear extensions of these methods with dominance and epistasis has been also considered (Nishio and Satoh, 2014; Jiang and Reif, 2015; Varona et al., 2018; Olatoye et al., 2019). However, recent development in the field of machine learning enable us to use profound nonlinear methods for the prediction of complex traits in breeding. Among the machine learning methods, deep learning (DL) methods received much attention due to their outstanding prediction properties (LeCun et al., 2015). Although improved accuracy can be questioned, many recent studies successfully applied deep learning for various genomic problems (Uppu et al., 2016; Bellot et al., 2018; Montesinos-López et al., 2018, 2019; Crossa et al., 2019; Liu et al., 2019; Pérez-Enciso and Zingaretti, 2019).

Often these learning methods are applied in a black-box manner and standard architectures that worked well in disciplines like natural language processing and computer vision are transferred to genomic prediction. Even though results are encouraging, interpretability remains an issue (Waldmann, 2018). However, as an exception, there is a study presenting an interpretable neural network model (see Zhao et al., 2021). Also, in this study we propose to design a domain specific learning system that is motivated by neural networks, but incorporates classical elements of lasso. The resulting algorithm is termed NeuralLasso, that is capable of incorporating higher order nonlinear interactions between contributing markers in the local neighborhood. Unlike the method of Zhao et al. (2021), our non-Bayesian approach is focusing on modeling high-order local interactions. In the terminology of neural networks, predictions are performed in a single layer and ℓ_1_ sparsity on the learned parameters is incorporated, hence the relation to classical lasso models. We compare the prediction accuracy of NeuralLasso with the most commonly used GP methods such as BayesA, BayesB, BL, GBLUP, and EN using the mouse and rice data sets.

## Models and Methods

###  Whole Genome Regression Model

Let us consider a standard genomic prediction model


(1) $$\begin{array}{l} y=X\beta +Zw+\epsilon . \end{array}$$

Here, *y* is a vector of observed phenotypes for *n* lines, β contains the fixed effects, *X* represents the incidence matrix for the fixed effects, *Z* = *Z*_*i*1_, *Z*_*i*2_, …*Z*_*ip*_ is the *n* × *p* (*p* is the number of markers) matrix for the genotypes coded as 0,1,2, *w* = (*w*_1_, *w*_2_….*w*_*p*_) is a column vector of marker effects and ϵ corresponds to the residual, following a normal distribution as $\epsilon \sim N\left(0,I\sigma_{e}^{2}\right)$. For simplicity, here we assume no fixed effects other than overall mean (note that it is possible to pre-correct fixed effects away from the phenotype before neural network analysis).

The number of markers usually exceeds the number of observations in genomic prediction problems and regularization is applied in order to obtain solution to Equation (1). A regularized regression function can be formulated as


(2) $$\hat{\beta },\hat{w}=\underset{\beta ,w}{\text{argmin}}\left[\sum_{i=1}^{n}{\left(y_{i}-X\beta -\sum_{j=1}^{p}Z_{ij}w_{j}\right)}^{2}+P(\lambda ,w)\right].$$

Here, the function *P*(λ, *w*) is the penalty function with regularization parameter λ ≥ 0. Least absolute shrinkage and selection operator (lasso) (Tibshirani, 1996) based on the penalty term called ℓ_1_-norm, which is the sum of the absolute coefficients and Ridge Regression based on the ℓ_2_-norm penalty which the sum of squared coefficients are the most commonly used regularized regression methods. The EN method which is a compromise between lasso and ridge regression penalties can be represented as:


(3) $$\begin{array}{l} \hat{\beta },ŵ=\underset{\beta ,w}{\mathrm{argmin}}\left\{\overset{n}{\underset{i=1}{\sum }}{\left(y_{i}-X\beta -\overset{p}{\underset{j=1}{\sum }}Z_{ij}w_{j}\right)}^{2}+\lambda \overset{p}{\underset{j=1}{\sum }}\left[\frac{1}{2}\left(1-\alpha \right)w_{j}^{2}+\alpha |w_{j}|\right]\right\} \end{array}$$

where 0 ≤ α ≤ 1 is the penalty weight. The EN penalty is controlled by α and when α = 1 EN is identical to lasso, whereas EN is equivalent to ridge regression when α = 0.

###  NeuralLasso

We design our model based on the underlying Equation (1). That is, given the genotypes in *Z*, instead of finding the matrix *w* we seek to find a parametrizable (nonlinear) mapping Λ_θ_, with parameters θ, such that


(4) $$\begin{array}{l} \Lambda_{\theta }\left(Z\right)=y. \end{array}$$

The question is how to construct such a mapping Λ_θ_ and what do the parameters of θ represent. In the following we aim to derive a model, that is motivated by neural networks, but follows the classical architecture of lasso as presented in Equation (3). For this purpose, we will shortly review classic neural network architectures.

#### Background on Neural Networks

The underlying premise of a neural network is to combine affine linear mappings and pointwise nonlinearities to construct a nonlinear mapping in a repeating multi-layered fashion (LeCun et al., 2015; Schmidhuber, 2015; Goodfellow et al., 2016). In its most general form we can write the main building block of a neural network for an input *z* ∈ ℝ^*n*^ (genotypes) and output *y* ∈ ℝ^*m*^ (phenotype) as


(5) $$\begin{array}{l} y=\varphi \left(Cz+b\right), \end{array}$$

where *C* ∈ ℝ^*m*×*n*^ is a linear transformation matrix, *b* ∈ ℝ^*m*^ an additive affine component, the so-called bias, and finally φ(·) a point-wise acting nonlinear function. A popular choice for this nonlinear function is given by the rectified linear unit ReLU(*x*): = max(*x*, 0). A multi-layered neural network would be now given as a repeated composition the blocks in Equation (5), where each block is called one layer. Nevertheless, in this work we concentrate on so-called shallow networks that consist only of one layer. The specific network architecture is now defined by the structure of the affine linear transformation *C*. The obvious choice of a dense matrix *C* is called a fully connected layer, as each data point in the input vector is related with each point in the output vector. Such a fully connected layer learns specific weights for each location in the input and hence is locally varying. Another option for the choice of linear mapping would be given by convolutions, if represented as matrices this would result in a sparse representation. Sparse representations are desirable, as they can be implemented efficiently and reduce the amount of parameters significantly. Nevertheless, the choice of convolutions as linear transformation is not optimal in our setting, as these are translationally invariant and hence do not encode any locality. In the following, we aim to design a transformation that is sparse, but does also encode locality to combine the strength of both.

###  Formulating NeuralLasso

The first important part is to define the underlying transformation given as the matrix *C* for our proposed model is based on the requirement to encode locality, while taking neighborhood relationships into account. For this purpose, we follow (Arridge and Hauptmann, 2019) and define a sparse subdiagonal matrix *C* ∈ ℝ^*p*×*p*^, where *p* is the number of markers, and a neighborhood of size *N*, such that the main diagonal and the *N* subdiagonals below and above are non-zero. That is for *N* = 0 we simply have a diagonal matrix and for *N* = 1 we have a tridiagonal matrix such as


(6) $$\begin{array}{l} C=\left(\begin{array}{ccccc} c_{0,1} & c_{1,1} & \; & \; & \;\\ c_{-1,2} & c_{0,2} & c_{1,2} & \; & \;\\ \; & \ddots & \ddots & \ddots & \;\\ \; & \; & c_{-1,n-1} & c_{0,n-1} & c_{1,n-1}\\ \; & \; & \; & c_{-1,n} & c_{0,n} \end{array}\right). \end{array}$$

Given the matrix *C* we could formulate a lasso problem that takes interactions in the local neighborhood into account by minimizing


(7) $$\begin{array}{l} Ĉ=\arg\underset{C}{\min}\overset{n}{\underset{i=1}{\sum }}{\left(y_{i}-\overset{p}{\underset{j=1}{\sum }}{\left(CZ_{i}^{T}\right)}_{j}\right)}^{2}+\lambda \overset{N}{\underset{j=-N}{\sum }}\overset{p}{\underset{i=1}{\sum }}|c_{j,i}|. \end{array}$$

Note, that for *N* = 0 no neighborhood relation is taken into account and the model reduces to the basic lasso scheme similar to Equation (3). As the above model in Equation (7) only considers linear interactions in the local neighborhood, we want to combine this sparse subdiagonal matrix with classical elements of neural networks, *i.e*., nonlinear activation functions and additional bias vectors to allow for nonlinear interactions, as outlined previously.

#### The Proposed Model for Local Epistatic Interactions

We will now consider the building block of a neural network as in Equation (5) for one layer, but consider multiplication with the subdiagonals of *C* separately to introduce nonlinear effects between neighboring loci. In the following, we will fix the neighborhood to *N* = 2, that is a neighborhood window of 5 loci. We will model the nonlinear interaction by a maximum thresholding using ReLU for the 3 central loci and no nonlinearity for the outer two loci. This way we enforce an interaction effect of the 5-neighborhood. Given the (sub)diagonal vectors $c_{i}\in {\mathbb{R}}^{p}$ for *i* = −2, …, 2 the non-linear parametrized model can be formulated as


(8) $$\begin{array}{l} \Lambda_{C}\left(Z_{j}\right)=\overset{2}{\underset{n=-2}{\sum }}\overset{p}{\underset{i=1}{\sum }}\varphi_{i}\left(c_{n,i}z_{i+n}+b_{n,i}\right), \end{array}$$

where *z*_*i*_ = 0 for *i* < 1 or *i* > *p*, and φ_*i*_(*x*) = ReLU(*x*) = max(*x*, 0) for *i* = −1, 0, 1 and φ_*i*_(*x*) = *x* otherwise. That is, if we write all terms down we get


(9) $$\begin{array}{l} \begin{array}{c} \Lambda_{C}\left(Z_{j}\right)=\overset{p}{\underset{i=1}{\sum }}\left(c_{-2,i}z_{i-2}+b_{-2,i}\right)+\left(c_{2,i}z_{i+2}+b_{2,i}\right)\\ \;+\text{ReLU}\left(c_{-1,i}z_{i-1}+b_{-1,i}\right)+\text{ReLU}\left(c_{0,i}z_{i}+b_{0,i}\right)\\ \;+\text{ReLU}\left(c_{1,i}z_{i+1}+b_{1,i}\right). \end{array} \end{array}$$

The resulting NeuralLasso then formulates as


(10) $$\begin{array}{l} \left\{Ĉ,\hat{b}\right\}=\arg\underset{\left\{C,b\right\}}{\min}\overset{n}{\underset{j=1}{\sum }}{\left[y_{j}-\Lambda_{C}\left(Z_{j}\right)\right]}^{2}+\;\lambda \overset{2}{\underset{n=-2}{\sum }}\overset{p}{\underset{i=1}{\sum }}\left(\left|c_{n,i}|+|b_{n,i}\right|\right). \end{array}$$

The parameters Ĉ and $\hat{b}$ can then be found by any suitable optimisation algorithm. ReLU functions were chosen, here, because of their ability to keep some of the linearity and introducing nonlinearity only by thresholding. Note that if all the ReLU activations are changed to linear functions then the model reduces to a sparse perceptron with biases (*i.e*., a single-layer neural network), which will be an overparametrized version of the lasso approach. We will shortly discuss our implementation in the next section.

For the final estimation, we are only left with estimating the penalty weight λ as in the classic lasso model. This can be achieved in a similar manner as used by Waldmann et al. (2019), here, we use a slightly modified bisection method and a single set of training data *i.e*., one realization of training and validation split. We then initialize a starting interval [*a, b*] for λ, chosen based on prior knowledge for the range of λ. We then compute the correlation coefficient for λ = *a, b*, *i.e*., the end points of the interval [*a, b*], and the mid point λ = *a* + *b*/2. Then, we identify the value for λ with the largest correlation coefficient. If it is one of the end points, we shift the interval around the end point, which becomes the new mid point. If, otherwise, the mid point has the highest correlation value, we keep the mid point, but halve the interval size. We then repeat the process for the new subinterval and compute the correlation for either one new point, if shifted, or two, if halved.

We note that for simplicity we have made here certain fixed choices and formulated NeuralLasso only for univariate continuous outcomes using fixed neighborhood size of 5, with ReLU as activation function and one layer. However, note that these are not arbitrary choices. As was stated earlier, ReLU was employed for its beneficial property of including linear functions as special case, if appropriate biases are learned. Some choices were found based on experimenting (*e.g*., neighborhood size of 5 provided good predictive performance) and some of the choices (use of ReLU and linear activation functions) are discussed more in the discussion section. We refer to the Appendix A for more a general formulation of NeuralLasso using variable neighborhood size and activation functions.

###  Example Analysis

In order to compare the prediction accuracy of different methods, we analyzed the rice field data which is publicly available at http://www.ricediversity.org/data/ and a heterogeneous stock mouse population [see Valdar et al. (2006) for more details]. We selected traits in these data sets, which cover many levels of heritabilities (ranging from 0.25 to 0.75) and arguably many different genetic architectures.

***Rice field data:*** The rice data set consists of 413 diverse accessions of *O. sativa* collected from 82 different countries (Zhao et al., 2011). The accessions were genotyped with single nucleotide polymorphism (SNP) markers and 33,569 SNPs were available for the analysis after excluding markers with minor allele frequency (MAF) > 0.05, duplicated markers and missing values > 20%. In this study, we analyzed the traits flowering time (FT) (in three different locations) and amylose content (AMY). The trait FT was measured in three different locations, the first location (ARK) was in Stuttgart, Arkansas, USA, the second one in Aberdeen (ABR) and the third location was Faridpur (FAD), Bangladesh (see Zhao et al., 2011 for more details). Out of the 413 lines, phenotypic informations were available for 371 lines in all three environments with the trait FT and 393 lines for the trait AMY. Genetic architecture underlying the trait (some traits are affected by many genes and some are by only few number of genes) is often play an important role in the prediction accuracy of different statistical methods. Thus we decided to consider two traits (FT and AMY) with different genetic architecture in this study. The narrow-sense SNP-heritabilities (*h*^2^) of the traits were 0.50, 0.70, 0.50, and 0.26 for the phenotypes AMY, ARK, ABR, and FAD, respectively. Here, *h*^2^ were estimated as: *h*^2^ = $\sigma_{g}^{2}/\left(\sigma_{g}^{2}+\sigma_{e}^{2}\right)$, where $\sigma_{g}^{2}$ and $\sigma_{e}^{2}$ are the genomic and residual variances, respectively. The variance components were estimated using GBLUP method.

***Heterogeneous stock mouse data:*** The mice data (see Valdar et al., 2006) consists of 1940 individuals with 10345 biallelic SNP markers after excluding markers with minor allele frequency (MAF) = 0.05 and missing values = 20%. In this study, we analyzed the trait “body weight,” which was measured at the age of 6 weeks. The narrow-sense SNP-heritability (*h*^2^) of the trait “body weight” was 0.58.

***Results:*** To demonstrate the superiority of our new approach, we compared the prediction accuracy (Pearson correlation coefficient between the observed and predicted phenotypes) of NeuralLasso with the most commonly used GP methods using the rice data set. The GP methods we are considering here are the GBLUP (Meuwissen et al., 2001), least absolute shrinkage and selection operator (lasso) (Tibshirani, 1996) and elastic net (EN) (Hoerl and Kennard, 1970). Also, Bayesian WGR models we choosed to consider here are the BL (Park and Casella, 2008), BayesA and BayesB (Meuwissen et al., 2001). Predictive abilities of BayesA, BayesB and BL were estimated using the R-package BGLR (Pérez and de los Campos, 2014). Whereas the predictive abilities of GBLUP and EN were estimated using the R-packages rrBLUP (Endelman, 2011) and glmnet (Simon et al., 2011), respectively. To estimate the predictive accuracy of NeuralLasso, the model was implemented in Python with TensorFlow and the scripts used in this study will be publicly available at: https://github.com/asHauptmann/NeuralLasso. Optimization was performed with the Adam algorithm and a cosine decay from 10^−3^ to 10^−5^ with 3,000 iterations, as batch size we used the full sample size.

In order to compare the prediction accuracies we used five-fold cross-validation (CV), for that we used 80% of the data as the training set and the remaining 20% as the validation set. To remove the influence of random partitions on the accuracy, we repeated the cross-validation procedure 50 times and took the mean value. Additionally, we also used the same training and validation sets with the different GP methods. In the analysis using BGLR, we used the default priors and considered 10,000 Markov Chain Monte Carlo iterations with a burn-in period of 3,000 iterations. For the EN estimation using glmnet, we set α to 0.33 in Equation (3) based on cross validation.

**Figure 1:**
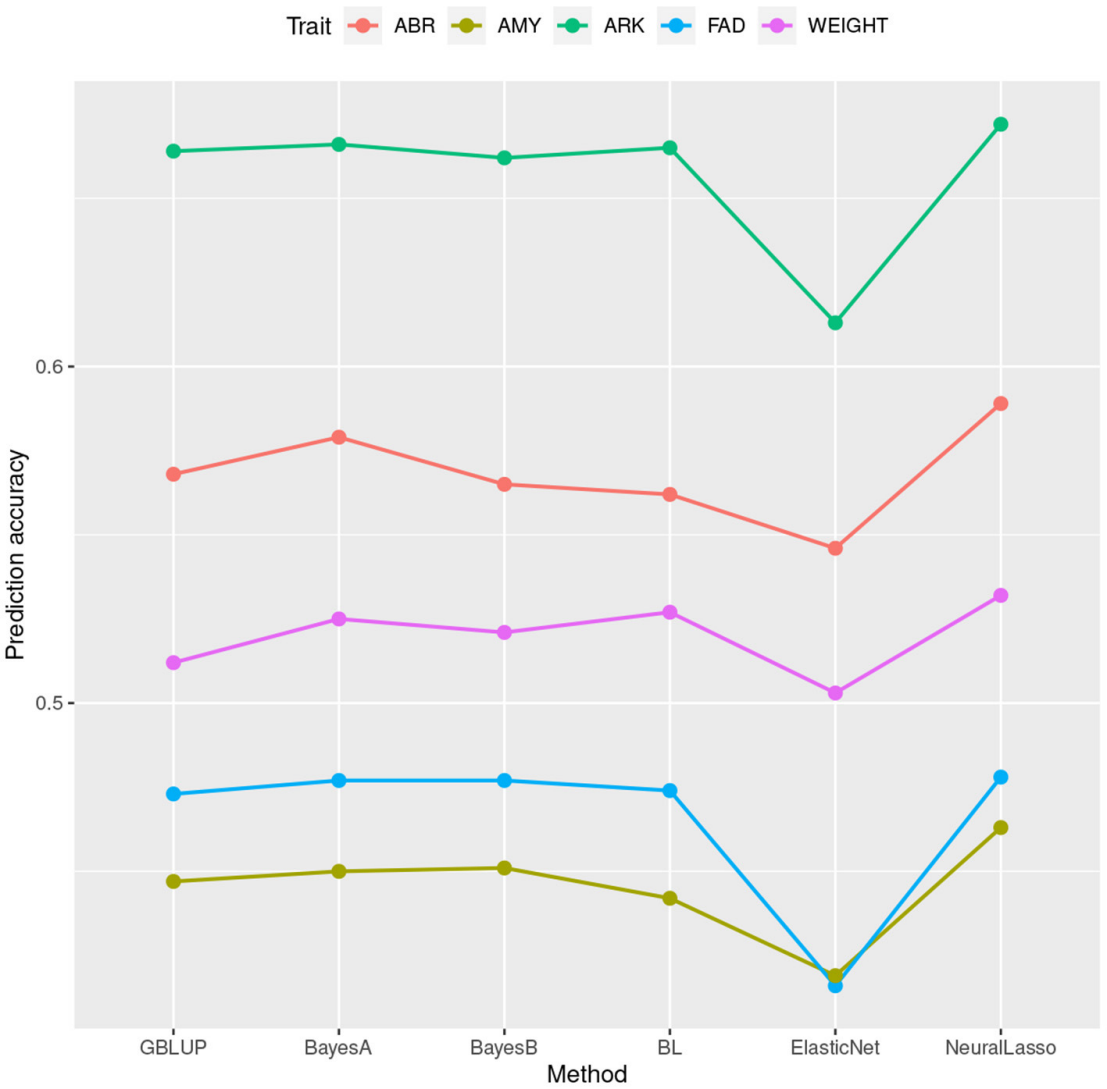
Mean prediction accuracy calculated based on 50 cross validations for different traits from the rice and mice data sets plotted against the corresponding estimation methods.

In Table 1, we can see the prediction accuracies of different methods in four traits (ARK, ABR, FAD, AMY) of rice data set, as well as trait body weight of mice data set. These traits together cover many levels of SNP-heritabilities. In traits ARK, ABR, and AMY of rice and trait body weight of mice, NeuralLasso seems to slightly outperform all the other methods, suggesting some role of local interactions in the genetic architecture of the trait. In trait FAD, the superior performance is much smaller and performance is practically the same with the GBLUP. This is likely due to much smaller SNP-heritability of the FAD than the other traits (see also Figure 1 which shows ordering of the methods in their prediction accuracies). Superiority of NeuralLasso method becomes clear also from here.

**Table 1:** Mean prediction accuracy based on 50 CV replicates using different approaches for the traits with rice (ARK, ABR, FAD, AMY) and mice (WEIGHT) data sets are shown along with the corresponding heritability (*h*^2^) estimate for the trait.

	**GBLUP**	**BayesA**	**BayesB**	**BL**	**ElasticNet**	**NeuralLasso**	***h* ^2^**
**Rice**							
ARK	0.664	0.666 (+0.30)	0.662 (−0.30)	0.665 (+0.15)	0.613 (−7.68)	0.672 (+1.20)	0.70
ABR	0.568	0.579 (+1.93)	0.565 (−0.52)	0.562 (−1.05)	0.546 (−3.87)	0.589 (+3.69)	0.50
FAD	0.473	0.477 (+0.84)	0.477 (+0.84)	0.474 (+0.21)	0.416 (−12.05)	0.478 (+1.05)	0.26
AMY	0.447	0.45 (+0.67)	0.451 (+0.89)	0.442 (−1.11)	0.419 (−6.26)	0.463 (+3.58)	0.50
**Mice**							
WEIGHT	0.512	0.525 (+2.53)	0.521 (+1.75)	0.527 (+2.92)	0.503 (−1.75)	0.532 (+3.90)	0.58

*Additionally, the percentage difference in prediction accuracy compared to the commonly used GBLUP estimation method is provided in the bracket*.

## Discussion

In this study, we have presented a shallow neural network method which takes into account higher order local epistatic interactions in each marker's neighborhood. In recent years, machine learning methods including deep learning (DL) methods have been widely considered for GP, however neural network methods perform similarly or worst to the classical linear methods (Azodi et al., 2019; Zingaretti et al., 2020; Montesinos-López et al., 2021). In this study with the tested cases, our proposed method seems to improve the prediction accuracy slightly over traditional methods. We believe that the accuracy of NeuralLasso will depend on the complexity of the trait. Unlike the traditional genomic prediction models which are able to account for the two-loci genome-wide interactions, NeuralLasso account for only the additive and higher order local epistatic genetic effects. Thus there is a reduced chance that the local epistatic genetic effect will disappear due to recombination and will be passed on to several generations (Akdemir and Jannink, 2015).

Even though, deep neural networks have been popular so far, size of learning data need to be large in many cases. We believe that more shallow networks like the one presented here may turn out to be useful and important in the future due to their more limited learning data size requirements.

In fact, our proposed model is not a single-layer neural network, *i.e*., a perceptron. In the classic perceptron the nonlinearity is applied after summation, in our case the nonlinearity is applied before to allow for nonlinear interactions. On the other hand, one could say it is only one layer, but with several channels, for each member of the neighborhood, that are combined nonlinearly. In summary, this is why we say the model is motivated by neural networks, but does not clearly fit in the classic notion of a neural network. Finally, that is why we also do not describe our model as a neural network, but as NeuralLasso, motivated by the design of neural networks.

We also tested the performance of NeuralLasso when changing all non-linear ReLU functions to linear ones (results not shown). In those experiments, the prediction accuracies of NeuralLasso method clearly dropped down in the rice data set but stayed at about the same level in the mice data [when ReLU functions in Equation (9) were replaced by linear functions]. This is well in line with what one expects to see in rice data (high level of epistasis) and in mice data (small or no level of epistasis). Therefore, the latter experiment arguably means that our NeuralLasso may also be capable of taking into account some other context-specific effects than only epistasis, because its predictive performance was so high in mice data set.

In this study, we only considered small genomic region, however, NeuralLasso can be adjusted to account for higher order genetic interactions in larger genomic region of interest, chromosome-wise or whole genome scale. Although this might be computationally challenging, it will be interesting to see if this turns out to be important in the future. In order to reduce the computational burden, one can also first perform a genome-wide association study (GWAS) and only account the regions of interest (*e.g.*, candidate gene regions) in NeuralLasso.

As in all genomic predictions, not any single statistical method is clearly superior in their prediction accuracy for all traits, but their performance depends on factors such as genetic architecture and heritability of the trait. However, NeuralLasso performance was found here to be promising and it is worth of considering in the future.

## Data Availability Statement

Publicly available datasets were analyzed in this study. This data can be found at: details are provided in the article.

## Author Contributions

BM, AH, JL, and MS: writing—review and editing and conceptualization. BM and AH: writing—original draft and formal analysis. All authors contributed to the article and approved the submitted version.

## Conflict of Interest

BM was employed by company Bayer CropScience. The remaining authors declare that the research was conducted in the absence of any commercial or financial relationships that could be construed as a potential conflict ofinterest.

## Publisher's Note

All claims expressed in this article are solely those of the authors and do not necessarily represent those of their affiliated organizations, or those of the publisher, the editors and the reviewers. Any product that may be evaluated in this article, or claim that may be made by its manufacturer, is not guaranteed or endorsed by the publisher.
