# Supplementary material for: NeuralLasso: Neural Networks Meet Lasso in Genomic Prediction
Source: Front Plant Sci. 2022 Apr 29;13:800161. doi: 10.3389/fpls.2022.800161 (PMC9100816; doi:10.3389/fpls.2022.800161)
Supplement: Supplementary file 1 [file Data_Sheet_1.pdf]

APPENDIX A: General formulation of Neural-Lasso

We can formulate a general form of NeuralLasso as follows. We have combined the subdiagonals from the neighbourhood matrix with the the neural network formulation, where the underlying operation is given by

$$\varphi_i(c_i \odot \tau_i(x) + b_i)).$$

Here $c_i, b_i \in \mathbb{R}^p$ are both vectors denoting a weighting from the matrix formulation and a bias, respectively. The weighting is applied by point-wise multiplication, denoted by $\odot$. Instead of writing the weighting as a matrix, we use a shift operator $\tau_j(x) : \mathbb{R}^p \to \mathbb{R}^p$, such that we can add a separate bias for each shift, or subdiagonal. The shift operator is defined by the index $j \in \mathbb{Z}$ for a vector x, for instance for $j = 1$ and $j = -1$ we have

$$\tau_1(x) = (0, x_1, x_2, \ldots, x_{n-1}) \text{ and } \tau_{-1}(x) = (x_2, x_3, \ldots, x_n, 0).$$

The nonlinearity φ_i can be chosen for each shift separately, in the following we consider two options for φ either as ReLU or no nonliearity, i.e. $\varphi(x) = x$. Note that the biases are essential to define the cut-off values for the ReLU operation.

The final model then combines the neighbourhood relations as follows. For a neighbourhood of size $N \geq 0$, we consider N samples to both the left and right of each index, multiply by the respective weights and add the bias, then the nonlinearities are applied and all $2N+1$ vectors are summed up. The final vector will then be summed to produce a single number as predicted value. In short, the model Λ_θ can be represented by a sum of the neighbourhood components by

$$A_\theta(x) = \mathbf{1}^T \left(\sum_{i=-N}^{N} \varphi_i \left(c_i \odot \tau_i(x) + b_i \right) \right)$$

With the parameter set $\theta = \{c_i, b_i\}$. Here $\mathbf{1}$ denotes the vector of ones in each entry. Then the final problem for the optimisation task is given by

$$\|A_\theta(x) - y\|_2^2 + \alpha \|\theta\|_1.$$

We note that the 1-norm of the parameter set is taken over all parameters independently, as if it would be a long vector, as written out in eq.(10).

The model considered in the study was obtained with the following choices:

$$N = 2,\ \varphi_{-1} = \varphi_0 = \varphi_1 = \text{ReLU},\ \text{and } \varphi_{-2} = \varphi_2 = Id,$$

which leads to the explicit formulation in eq.(9). That is a 5-neighbourhood connection with a positivity condition on the central and closest neighbours.
